# Subgroup analysis of steadily increased trends in medullary thyroid carcinoma incidence and mortality in the USA, 2000–2020: a population-based retrospective cohort study

**DOI:** 10.1530/ERC-23-0319

**Published:** 2024-03-18

**Authors:** Zixia Tao, Xianzhao Deng, Bomin Guo, Zheng Ding, Youben Fan

**Affiliations:** 1 Department of General Surgery, Thyroid and Parathyroid Center, Shanghai Sixth People's Hospital Affiliated to Shanghai Jiao Tong University School of Medicine, Shanghai, China

**Keywords:** medullary thyroid carcinoma, incidence, mortality, SEER

## Abstract

The incidence rate of medullary thyroid carcinoma (MTC) continues to grow, along with its mortality rate in the USA. However, the subgroup trends in MTC have not yet been established. This population-based retrospective cohort study was based on the Surveillance, Epidemiology, and End Results (SEER) 17/12 registry database. Subgroup analysis was performed through clinicopathological and treatment-related characteristics. Annual average percentage change (AAPC) was calculated using joinpoint regression analysis. A total of 3833 MTC patients and 536 death cases were diagnosed in the SEER database. Between 2000 and 2019, the incidence (AAPC = 1.64) and mortality (AAPC = 3.46) rates of MTC continued to rise. Subgroup analysis showed the proportion of elderly patients (65–84 years) gradually increased in incidence between 2000 and 2020. Patients with early-stage tumors, such as tumors ≤20 mm, showed the same trends. Aspects of treatment, the implementation rate of total thyroidectomy (AAPC = 0.38) and lymph node dissection (AAPC = 1.06) also increased persistently in almost all of the age subgroups. The incidence and mortality of MTC consistently increased from 2000 to 2019. Subgroup analysis indicated a significant increase in elderly patients and early-stage patients, and more attention should be paid to the management of these increased subgroups.

## Introduction

Medullary thyroid carcinoma (MTC) originates from parafollicular cells of the thyroid and is characterized by the secretion of calcitonin and carcinoembryonic antigen. Unlike differentiated thyroid carcinoma, endocrine suppressive therapy and iodine-131 treatment are ineffective against it, and surgery is the only potential approach (Wells *et al.* 2015). MTC represents approximately 2% of thyroid malignancies; however, it accounts for approximately 8% of thyroid malignancy mortality, due to its high degree of malignancy (Lim *et al.* 2017).

Over the past few decades, the incidence of thyroid cancer in the USA has increased significantly (Lim *et al.* 2017, Megwalu & Moon 2022). This may be due to the growth in the diagnosis of microcarcinoma (Haugen *et al.* 2016, Pizzato *et al.* 2022). After the implementation of fine needle aspiration (FNA) on small thyroid nodules was restricted, the incidence of thyroid cancer and papillary thyroid carcinoma slowed down after 2009 and began to decline after 2014, but the incidence of MTC continued to rise persistently between 2000 and 2018 (Kitahara & Sosa 2016, Lim *et al.* 2017, Megwalu & Moon 2022). The continued increase in MTC incidence may raise new challenges for management. Randle *et al.* summarized the subgroup trends of MTC from 1983 to 2012 in the USA. They divided the study into three time periods, 1983–1992, 1993–2002, and 2003–2012, and observed an increase in both proportion of microcarcinoma and patients who underwent total thyroidectomy (Randle *et al.* 2017). However, there has been a lack of research investigating more detailed subgroup trends in MTC incidence and mortality in recent years in the USA.

In the present study, we updated the latest trends in MTC and demonstrated the detailed subgroup trends for the first time, based on the Surveillance, Epidemiology, and End Results (SEER) program. The results of our study indicate that the incidence and mortality of MTC in the USA is still rising, and the increase in elderly patients and early-stage patients may raise new challenges in the management of MTC.

## Materials and methods

### Data source

Age-adjusted incidence and incidence-based mortality (IBM) data were extracted through the Surveillance, Epidemiology, and End Results (SEER) program. Incidence was based on the SEER-17 Registry database (2000–2020), and IBM was based on the SEER-12 Registry database (1992–2020). The SEER program included 17 high-quality population-based registries, covering 28% of the US population. Approval of this study was waived by our institution’s ethics review board.

To calculate the incidence and mortality rates of MTC, the cohort was selected by the following variables. ‘Primary site label’ variable was set to C73.9, and pathological types were selected using the ‘ICD3-O-3’ variable: MTC (8345, 8510-8513). Only microscopically confirmed cases were included and cases of autopsy and death certificates only were excluded by the ‘Diagnostic Confirmation’ and ‘Type of reporting source’ variables. The calculation of mortality rates only included first-matching record cases. All rates were age-adjusted to the 2000 US standard population.

Subgroups mainly included demographic characteristics (gender, race, age at diagnosis/death), clinicopathological characteristics (tumor size, extrathyroid invasion, N stage, M stage), and surgical procedures (surgery of primary site and regional lymph nodes). Of these, surgery of regional lymph nodes only included patients who underwent surgery. Gender, age, and race were selected using the ‘Sex’, ‘Age recode with <1-year-olds’, and ‘Race recode (W, B, AI, API)’ variables. Clinicopathological characteristics were mainly selected and classified using ‘EOD-10 (1988-2003)’, ‘CS Tumor (2004-2015)’, ‘Derived SEER Combined (2016-2017)’, and ‘EOD Primary tumor (2018+)’. The surgery type of the primary site was screened by ‘Site-specific surgery (1973-1997)’ and ‘Rx Summ-Surg Prim site (1998+)’ variables. Surgery of regional lymph nodes was classified by the ‘Regional nodes examined (1988+)’ variable. Survival status was defined using the ‘Survival Month’ and ‘Vital Status recode’ variables.

### Statistical analyses

Incidence, incidence-based mortality rates, and characteristic information were obtained through SEER*Stat version 8.4.2 (National Cancer Institute). Annual percentage change (APC), annual average percentage change (AAPC), and 95% confidence intervals (CIs) were calculated by Joinpoint Regression Program version 5.0.0 (National Cancer Institute). Incidence and mortality data, extracted from the SEER database, were imported into the Joinpoint program, where joinpoints (nodes with changing trends) and APC/AAPC were calculated utilizing an optimized log-linear regression model. The significance of trends in incidence and mortality was determined by assessing if there was a statistically significant difference between APC/AAPC and 0, using the *t*-test. To reduce the impact of COVID-19 on incidence and mortality trends, 2020 was excluded from trend calculations (subtype analysis did not exclude 2020). All subgroups were calculated in percentage to minimize the impact of change in total numbers. In some years, where specific subgroups had zero deaths, they were defined as 0.01 to ensure the normal operation of the Joinpoint program. All tests were two-tailed with *α* defined as 0.05.

## Results

### Clinicopathologic features of the patients with MTC in the SEER database (2000–2020)

A total of 3833 MTC patients were identified from the SEER-17 database between 2000 and 2020. Among 3833 MTC patients, 57.8% were females and 42.5% were diagnosed at 45–64 years; 50.4% patients had tumor size ≤20 mm, 56.9% patients were without lymph node metastasis, and 10.3% patients had distant metastasis when diagnosed. Moreover, 84.3% patients received total thyroidectomy, and 75.2% patients received lymph node dissection (Table 1).

**Table 1:** Clinicopathologic and treatment-related characteristics of patients with medullary thyroid carcinoma from 2000 to 2020.

Characteristics	No. of patients (*n*)	Percentage (%)
Sex		
Male	1616	42.2
Female	2217	57.8
Race		
White	3209	83.7
Black	321	8.4
Others	303	7.9
Age at diagnosis (years)		
0–19	147	3.8
20–44	888	23.2
45–64	1631	42.5
65–84	1096	28.6
≥85	71	1.9
Tumor size (mm) (*n* = 3519)		
0–10	883	25.1
11–20	889	25.3
21–39	1042	29.6
≥40	705	20.0
Invasion (*n* = 3586)		
Intrathyroid	2861	79.8
Extrathyroid	432	12.0
Locally advanced	293	8.2
N stage (*n* = 3631)		
N0	2065	56.9
N1a	508	14.0
N1b	736	20.2
N1x	322	8.9
M stage (*n* = 3737)		
M0	3352	89.7
M1	385	10.3
Surgery in primary site (*n* = 3768)		
No surgery	335	8.9
Lobectomy	258	6.8
Total thyroidectomy	3175	84.3
Surgery in regional lymph node (*n* = 3418)		
No LND	849	24.8
LND	2569	75.2

LND, lymph node dissection.

### Incidence and mortality trends of MTC in the USA (2000–2019)

Between 2000 and 2020, a total of 3833 cases of MTC were diagnosed in the SEER-17 registry database, with a total of 536 death cases of MTC in the SEER-12 registry database. The number of incidence and death cases steadily increased from 660 and 79 cases in 2000–2004 to 1102 and 190 cases in 2015–2019.

**Figure 1:**
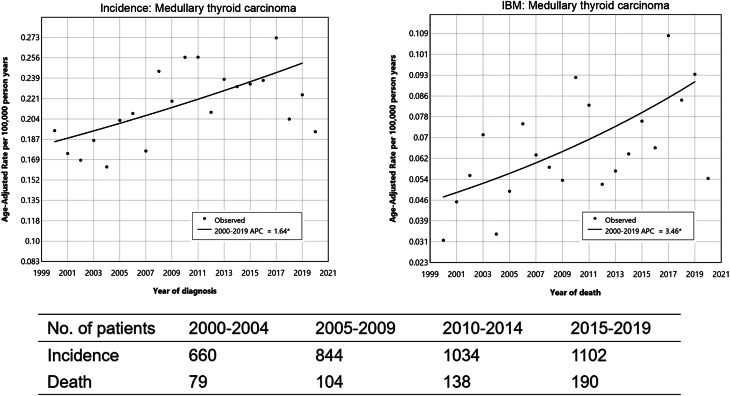
Incidence and incidence-based mortality rate of medullary thyroid carcinoma (2000–2019). IBM, incidence-based mortality; APC, annual percent change.

Joinpoint regression analysis showed the incidence (APC = 1.64) and mortality (APC = 3.46) rates of MTC continued to rise from 2000 to 2019 in the USA. The incidence and mortality rate were 0.225 and 0.094 per 100,000 person-years in 2019, respectively (Fig. 1).

### Subgroup analysis of incidence and mortality trends of MTC in the USA (2000–2020)

**Figure 2:**
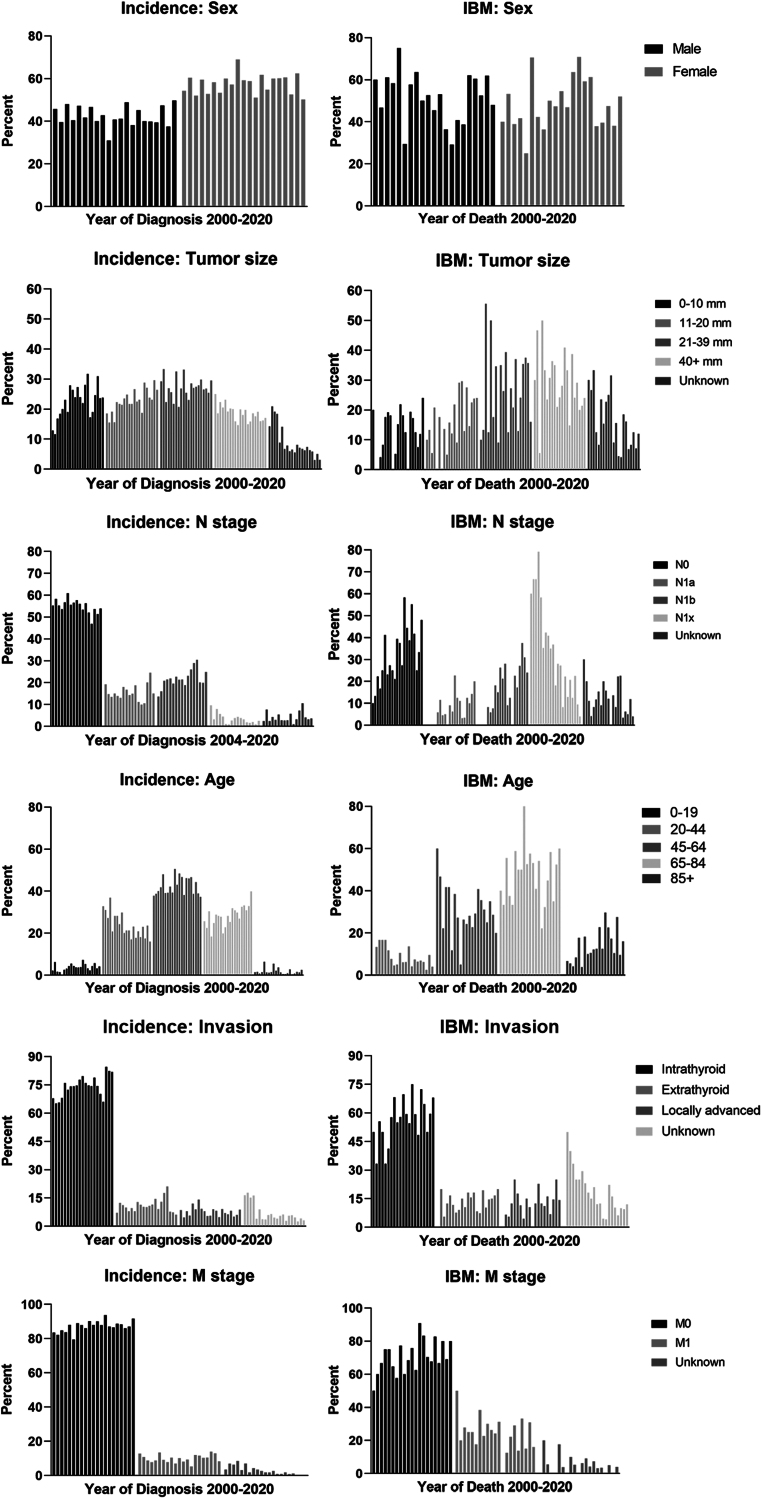
Clinicopathological subgroup analysis of incidence and incidence-based mortality rate of medullary thyroid carcinoma (2000−2020).

**Figure 3:**
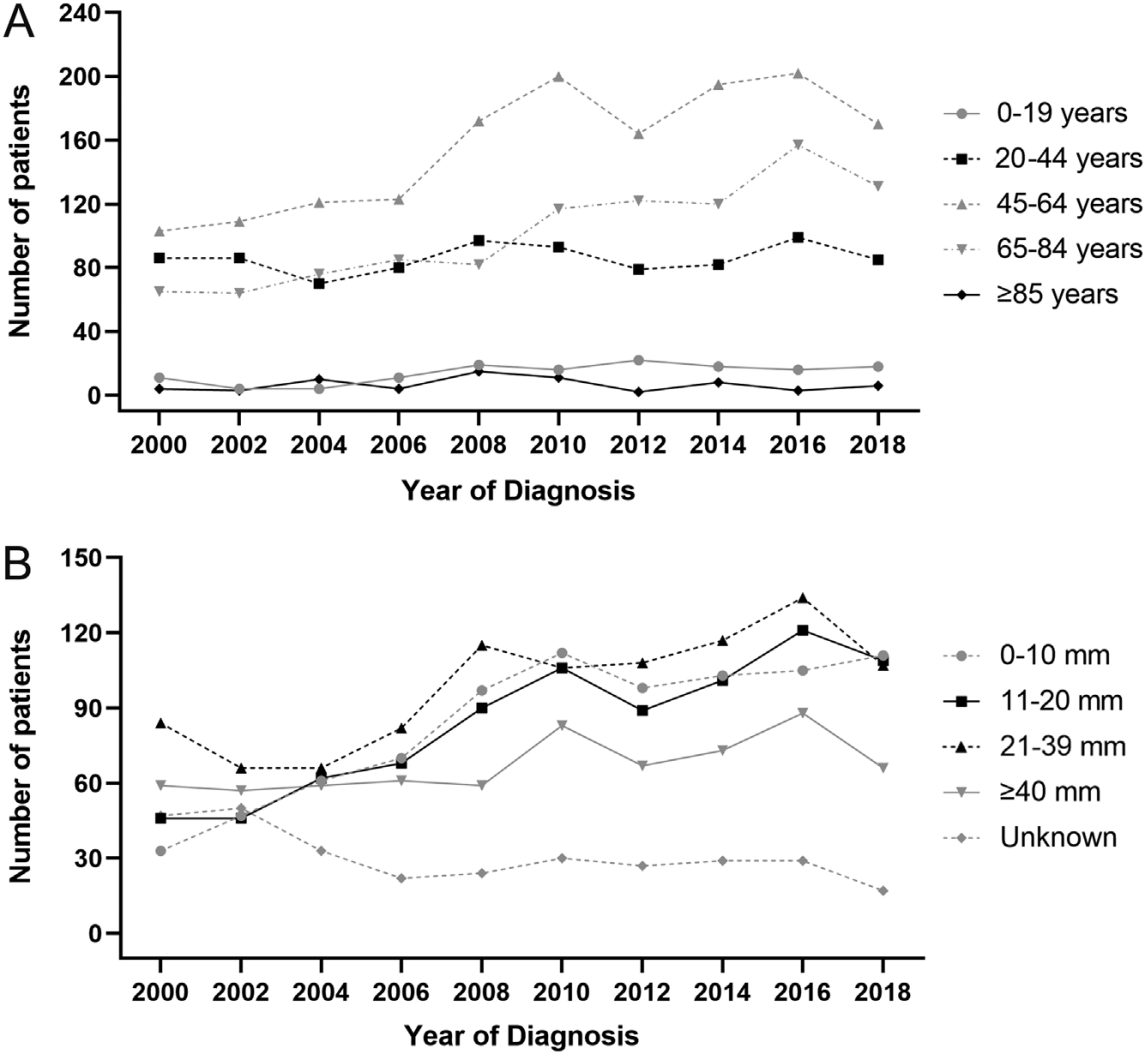
Number of medullary thyroid carcinoma patients in age (A) and tumor size (B) subgroups every 2 years from 2000 to 2019.

Subgroup analysis showed that the subgroup trends significantly changed from 2000 to 2020 in the United States (Table 2). In terms of incidence, the proportion of patients aged 20–44 years (AAPC = −2.90, *P* < 0.001) decreased significantly. Conversely, the proportion of patients aged 65−84 years grew (AAPC = 1.87, *P* < 0.001). Moreover, there was a considerable increase in the 0−10 mm subgroup (AAPC = 3.43, *P* = 0.047), the 11−20 mm subgroup (AAPC = 1.94, *P* < 0.001), the intrathyroid subgroup (AAPC = 0.77, *P* = 0.002), and the M0 subgroup (AAPC = 0.32, *P* = 0.011). A similar phenomenon was observed in the subgroup distribution of mortality. The proportion of ≥85 years subgroup (AAPC = 4.06, *P* = 0.043), 11−20 mm subgroup (AAPC = 2.91, *P* = 0.027), intrathyroid subgroup (AAPC = 1.47, *P* = 0.031), and M0 subgroup (AAPC = 1.03, *P* = 0.039) significantly grew from 2000 to 2020. The bar graph in Figure 2 shows the subgroup trends in incidence and mortality from 2000 to 2020. Figure 3 displays the number of patients in age and size subgroups from 2000 to 2019 every 2 years. The number of patients in subgroups showed the same trends mentioned previously: the number of patients in 45−84 years and 0−20 mm subgroups increased from 2000 to 2019, while the 0−44 years and ≥40 mm subgroups remained stable.

**Table 2:** Subgroup analysis on clinicopathological characteristics of medullary thyroid carcinoma incidence and incidence-based mortality from 2000 to 2020.

Characteristics	Incidence	*P*	IBM	*P*
	AAPC (95% CI)		AAPC (95% CI)	
Sex				
Male	–0.04 (–0.83, 0.76)	0.916	–0.68 (–2.12, 0.78)	0.341
Female	0.10 (–0.54, 0.73)	0.753	0.46 (–1.45, 2.40)	0.622
Age at diagnosis (years)				
0–19	0.18 (–2.56, 3.00)	0.893	–^b^	
20–44	–2.90 (–4.03, –1.75)	**<0.001**	–4.89 (–7.25, –2.47)	**<0.001**
45–64	–0.27 (–1.90, 1.39)	0.748	–3.55 (–7.52, 0.59)	0.092
65–84	1.87 (0.96, 2.80)	**<0.001**	0.23 (–1.78, 2.28)	0.815
85+	2.91 (–10.93, 18.90)	0.697	4.06 (0.14, 8.13)	**0.043**
Tumor size (mm)				
0–10	3.43 (0.05, 6.93)	**0.047**	0.25 (–1.86, 2.41)	0.805
11–20	1.94 (1.03, 2.85)	**<0.001**	2.91 (0.37, 5.52)	**0.027**
21–39	–0.16 (–1.10, 0.79)	0.731	–2.15 (–4.80, 0.57)	0.114
40+	–1.60 (–2.37, –0.83)	**<0.001**	–2.47 (–4.39, –0.50)	**0.017**
Invasion				
Intrathyroid	0.77 (0.32, 1.23)	**0.002**	1.47 (0.15, 2.84)	**0.031**
Extrathyroid	–3.71 (–10.50, 3.60)	0.311	0.62 (–1.41, 2.69)	0.533
Locally advanced	–1.72 (–3.95, 0.56)	0.130	0.61 (–2.43, 3.73)	0.684
N stage^a^				
N0	–0.60 (–1.12, –0.09)	**0.025**	2.26 (–1.18, 5.83)	0.182
N1a	1.43 (–0.88, 3.78)	0.209	2.95 (–2.30, 8.48)	0.253
N1b	2.39 (0.63, 4.17)	**0.010**	4.15 (0.48, 7.95)	**0.029**
N1x	–9.30 (–12.42, –6.08)	**<0.001**	–7.03 (–9.72, –4.26)	**<0.001**
M stage				
M0	0.32 (0.08, 0.57)	**0.011**	1.03 (0.06, 2.02)	**0.039**
M1	0.51 (–0.98, 2.02)	0.484	–2.85 (–9.73, 4.56)	0.441

Values in bold indicate statistical significance.

^a^N stage’s AAPC is calculated by cases diagnosed from 2004 to 2020 (incidence) and died from 2005 to 2020 (IBM) as SEER record before 2003 could not distinguish N1a and N1b; ^b^Only 1 death case in 0–19 group, failed to calculate.

AAPC, average annual percent change; CI, confidence interval; IBM, incidence-based mortality.

### Subgroup analysis of treatment-related characteristics trends of MTC in the USA (2000−2020)

**Figure 4:**
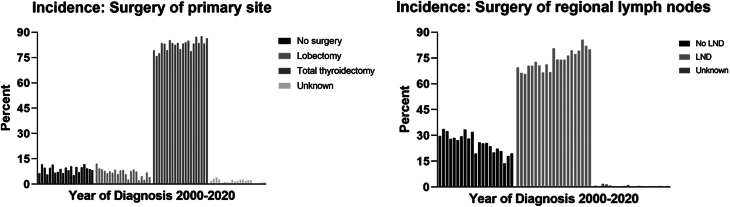
Treatment-related subgroup analysis of incidence and incidence-based mortality rate of medullary thyroid carcinoma (2000–2020). LND, lymph node dissection.

The trend of treatment-related characteristics showed that patients who underwent total thyroidectomy (AAPC = 0.38, *P* = 0.003) and those who underwent lymph node dissection (AAPC = 1.06, *P* < 0.001) significantly increased from 2000 to 2020. In particular, the proportion of patients who chose no surgery remained stable (AAPC = 0.0, *P* = 0.994) (Table 3). The bar graph in Figure 4 shows the trends in treatment-related characteristics from 2000 to 2020. Table 4 presents the surgical trends across various age groups. Almost all groups exhibited a slight increase in the rate of total thyroidectomy and lymph node dissection (LND), and a corresponding decrease in lobectomy and non-LND rates. Notably, patients over 85 years experienced a substantial increase in surgery rates, escalating from 63.6% to 77.8%.

**Table 3:** Subgroup analysis on treatment-related characteristics of medullary thyroid carcinoma incidence from 2000 to 2020.

Characteristics	Incidence	*P*
	AAPC (95% CI)	
Surgery in primary site		
No surgery	0.01 (–1.53, 1.56)	0.994
Lobectomy	–2.79 (–4.45, –1.09)	**0.003**
Total thyroidectomy	0.38 (0.15, 0.60)	**0.003**
Surgery in regional lymph node		
No LND	–2.66 (–3.60, –1.71)	**<0.001**
LND	1.06 (0.74, 1.38)	**<0.001**

Values in bold indicate statistical significance.

AAPC, average annual percent change; CI, confidence interval; LND, lymph node dissection.

**Table 4:** Trends in surgical treatment by different age groups from 2000 to 2020.

Age (years)	Year of diagnosis	Surgery in primary site, *n* (%)^a^			Surgery in regional lymph nodes, *n* (%)^b,c^	
		No surgery	Lobectomy	Total thyroidectomy	No LND	LND
0–19	2000–2010	0 (0.0)	1 (1.8)	53 (93.0)	12 (22.2)	41 (75.9)
	2011–2020	3 (3.3)	4 (4.4)	82 (91.1)	19 (22.1)	67 (77.9)
						
20–44	2000–2010	27 (5.8)	27 (5.8)	400 (86.0)	101 (23.7)	324 (75.9)
	2011–2020	32 (7.6)	14 (3.3)	371 (87.7)	57(14.8)	328(85.2)
						
45–64	2000–2010	50 (6.9)	58 (8.0)	604 (83.8)	171 (25.8)	486 (73.4)
	2011–2020	80 (8.8)	46 (5.1)	772 (84.8)	173 (21.1)	643 (78.6)
						
65–84	2000–2010	53 (12.2)	50 (11.5)	319 (73.7)	146 (39.6)	222 (60.2)
	2011–2020	68 (10.3)	46 (6.9)	537 (81.0)	151 (25.9)	429 (73.6)
						
>85	2000–2010	16 (36.4)	3 (6.8)	25 (56.8)	13 (46.4)	15 (53.6)
	2011–2020	6 (22.2)	3 (11.1)	18 (66.7)	6 (28.6)	14 (66.7)

^a^The percentage calculation includes patients with unknown treatment, but this group of patients is not shown in the table;^ b^The LND-related calculations excluded patients opting no surgery and patients with unknown treatment (*n* = 3433); ^c^The percentage calculation includes patients with unknown LND, but this group of patients is not shown in the table.

## Discussion

There have been several previous studies which reported the increased or steady incidence trends of MTC in the USA (Lim *et al.* 2017, Randle *et al.* 2017, Megwalu & Moon 2022). However, as far as we know, there was no study focused on the detailed subgroup trends of MTC in recent years. In the present study, we provide an updated analysis of the current trends in MTC and demonstrate the detailed subgroup trends for the first time. Our study results indicate that the incidence and mortality of MTC in the USA are increasing persistently, and the escalating trends in elderly patients and patients diagnosed at an early stage may pose new challenges to effective management strategies for MTC.

Megwalu & Moon found that 2014 was the joinpoint where the incidence rate of thyroid cancer began to decline in the USA. At the same time, they analyzed the histological subtype of thyroid cancer and found that the incidence and mortality rate of MTC continued to rise from 2000 to 2018, but mortality did not show statistical significance (Megwalu & Moon 2022). Our results verify the increased trends in incidence and mortality rate in MTC from 2000 to 2019. In particular, the results show that the increased trend of mortality rate showed statistical significance for the first time, indicating that real deaths might be increasing.

Furthermore, we conducted a detailed subgroup analysis on MTC from 2000 to 2020 and found that the increased subgroups of MTC incidence were mainly in patients with early-stage tumors such as tumor size ≤20 mm, intrathyroid, and M0 stage, which was more significant than the slight trends from 1973 to 2000 (Kebebew *et al.* 2005). Similarly, Lim *et al.* reported that the incidence of papillary thyroid carcinoma was mainly increased by early-stage patients, especially in patients with tumor size ≤10 mm (Lim *et al.* 2017). Some studies indicated that the increase in early-stage tumors may be caused by the frequent use of ultrasound and FNA screening. On the contrary, some scholars believe that the true incidence is increasing due to the continuous increase in mortality (Ahn *et al.* 2014, Zevallos *et al.* 2015, Lim *et al.* 2017). Our results showed that the mortality of both MTC patients and early-stage MTC patients grew significantly, which might suggest the underlying true increase in incidence.

In particular, our study finds that there has been a significant change in the distribution of diagnosis age from 2000 to 2020. In terms of the incidence rate, the proportion of patients diagnosed at 20–44 years (AAPC = –2.90) decreased, while those diagnosed at 65–84 years significantly increased (AAPC = 1.87). Moreover, for the mortality rate, the proportion of patients dead at 20–64 years (AAPC = –4.89 in patients dead at 20–44, AAPC = –3.55 in patients dead at 45–64) declined, and those dead after 85 years (AAPC = 4.06) increased. There were no reports of this phenomenon before. We speculated that the 8th AJCC’s TNM staging defined the patients younger than 55 years old as early-stage patients in differentiated thyroid carcinoma, which might lead young patients with thyroid nodules to choose active monitoring and reduce the percentage of young patients in the MTC patient group (Haugen *et al.* 2016, Grani *et al.* 2020). However, the reason for the increased incidence of elderly patients is still unknown, and this phenomenon may raise new challenges in the management of MTC. Elderly patients tend to die of diseases such as stroke, high blood pressure, and other cardiovascular illnesses, and previous studies have shown that patients older than 65 years is an independent prognostic risk factor for MTC patients, while the effectiveness of surgery in elderly patients has not been proved (Opsahl *et al.* 2019, Sahli *et al.* 2021). Finding the best balance between complications, survival rate, and living quality is a problem that needs to be solved in the future.

In addition, our study also conducted an analysis of treatment-related characteristics. The 2015 American Thyroid Association guideline for MTC recommended that total thyroidectomy with bilateral central lymph node dissection was the standard surgical approach for MTC patients. Similar to Frisco’s study, which found that the rate of standard surgery increased from 63.0% in 2000–2009 to 76.0% in 2016–2018 in the USA, we observed that the implementation of standard recommended surgical procedures was steadily growing from 2000 to 2020 (Frisco *et al.* 2023). A subgroup analysis stratified by age was conducted to explore the surgical trends in different age groups, and we found that all age subgroups, except patients diagnosed at 0–19 years, exhibited higher total thyroidectomy rates in 2011–2020 compared to 2000–2010. Notably, in patients over 85 years, a substantial increase was observed in the percentage of patients who opted for surgery, rising from 63.6% to 77.8%. Surprisingly, a slight decline in surgery rates was noted in patients aged 20–44 and 45–64. Given the favorable prognostic impact of surgery in most patients, further research should be conducted to explore the driving factors for opting to forgo surgery in these patients (Hirsch *et al.* 2018, Elsamna *et al.* 2021, Shao *et al.* 2022, Wilhelm *et al.* 2023).

Notably, the observed increases in the incidence and mortality of MTC, as well as the subgroup distribution trends identified in our study, are specific to the USA and cannot be generalized to global trends. Epidemiological data from Canada and Norway also demonstrate a similar rise in the incidence of MTC (Opsahl *et al.* 2019, Ellison & Bushnik 2020). However, in China, the UK, and South Korea, the incidence trend of MTC has remained relatively stable or has even decreased (Du *et al.* 2018, Ahn *et al.* 2020).

There are several limitations to this study. First, due to the descriptive nature of our study, we can only speculate on the observed trends and subtype changes of MTC. Although SEER-17 Registries cover 28% of the US population, there are still limitations. Second, in the SEER program, the N stage of MTC from 1988 to 2003 cannot distinguish ‘N1a’ and ‘N1b’ from ‘N1x’, so the calculations including ‘N1a’ and ‘N1b’ might have some biases. Third, the SEER database lacked genetic information to distinguish hereditary MTC and sporadic MTC, which have different clinicopathological features, recommended surgical procedures, and prognosis (Khurana *et al.* 2004, Machens & Dralle 2012). Based on the 2015 ATA guideline, hereditary MTC accounts for approximately 25% of total MTC patients (Wells *et al.* 2015). However, a recent multicenter cohort study in Israel showed that the proportion of hereditary MTC declined from 41.9% between 1981 and 1995 to 8.2% between 2006 and 2016 (Hirsch *et al.* 2018). The latest subgroup trends in the distribution of gene information of MTC patients in the USA needs to be updated in further studies to better guide treatment. Lastly, some serum indicators such as preoperative/postoperative calcitonin levels, carcinoembryonic antigen levels, and pathological characteristics such as necrosis and Ki67 proliferation index are important in the diagnosis and treatment of MTC. However, the SEER database lacked detailed information mentioned previously (Raue *et al.* 2021, Xu *et al.* 2022). Further large sample retrospective cohort studies and epidemiological reports with detailed information should be conducted in the future.

## Conclusion

Analysis of the SEER database revealed that the incidence and mortality rates of MTC steadily increased in the USA. Subgroup analysis indicated a significant increase in elderly patients and early-stage patients, which may raise new challenges in the management of MTC. However, further study should be conducted to verify the trends.

### Declaration of interest

The authors declare that there is no conflict of interest that could be perceived as prejudicing the impartiality of the study reported.

### Funding

The authors declare that they received no financial support or sponsorship for the study.

### Author contribution statement

Tao, Zixia: data curation; formal analysis; methodology; writing – original draft; Deng, Xianzhao: validation; writing – review and editing; Guo, Bomin: investigation; supervision; Ding, Zheng: investigation; Fan, Youben: conceptualization; supervision.
